# The Changes in the Severity of Deep Neck Infection Post-UPPP and Tonsillectomy in Patients with OSAS

**DOI:** 10.3390/life12081196

**Published:** 2022-08-05

**Authors:** Pin-Ching Hu, Liang-Chun Shih, Wen-Dien Chang, Jung-Nien Lai, Pei-Shao Liao, Chih-Jaan Tai, Chia-Der Lin, Hei-Tung Yip, Te-Chun Shen, Yung-An Tsou

**Affiliations:** 1 Department of Otolaryngology-Head and Neck Surgery, China Medical University Hospital, Taichung 404332, Taiwan; 2 Department of Otolaryngology-Head and Neck Surgery, Asia University Hospital, Taichung 40002, Taiwan; 3 Graduate Institute of Biomedical Sciences, China Medical University, Taichung 40402, Taiwan; 4 Department of Sport Performance, National Taiwan University of Sport, Taichung 404401, Taiwan; 5 School of Chinese Medicine, College of Chinese Medicine, China Medical University, Taichung 40402, Taiwan; 6 School of Medicine, China Medical University, Taichung 40402, Taiwan; 7 Department of Health Services Administration, China Medical University, Taichung 40402, Taiwan; 8 Management Office for Health Data, China Medical University Hospital, Taichung 404332, Taiwan; 9 Division of Pulmonary and Critical Care Medicine, Department of Internal Medicine, China Medical University Hospital, Taichung 40447, Taiwan

**Keywords:** deep neck infection, uvulopalatopharyngoplasty, obstructive sleep apnea syndrome, National Health Insurance Research Database

## Abstract

The main aim of this study is to compare the incidence rate and severity of deep neck infection (DNI) in patients post-UPPP+ T (uvulopalatopharyngoplasty plus tonsillectomy) and without UPPP+ T. We utilized the data derived from the Longitudinal Health Insurance Database (LHID) of the National Health Insurance Research Database (NHIRD) in Taiwan from 1 January 2000 to 31 December 2012. Patients who had undergone combined UPPP and tonsillectomy were selected using National Health Insurance (NHI) surgical order. Patients with DNI were selected using International Classification of Diseases (ICD-9-CM) code. A logistic regression model was applied for risk analysis. There were 1574 patients in the UPPP+ T cohort, and 6,296 patients who did not undergo combined UPPP and tonsillectomy for the control group. Our analysis showed that patients with an obstructive sleep apnea syndrome (OSAS) history constitute 76.1% (*n* = 1198) of the UPPP+ T cohort. Compared to the control group, there was no significantly increased incidence rate of DNI after UPPP+ T within 1–60 months. Patients undergoing combined UPPP and tonsillectomy had a lower intubation rate for DNI, with an adjusted odds ratio of 0.47 (95% CI = 0.32–0.69). The combined UPPP and tonsillectomy does not increase the risk of DNI within 1–60 months. Furthermore, combined UPPP and tonsillectomy can reduce the severity for DNI by decreasing the intubation rate and length of hospitalization.

## 1. Introduction

Deep neck infection (DNI) is a severe but treatable infection of deep cervical spaces, featuring rapid disease progression and many life-threatening complications [1]. Potential causes of DNI include odontogenic infections, salivary origin infections, pharyngitis, tonsillitis, cervical lymphadenitis, and trauma to the head and neck. There were several deep cervical spaces frequently related to tonsillitis, including peritonsillar space, submandibular space, parapharyngeal spaces, carotid spaces, and retropharyngeal space. Tonsillitis related peritonsillar abscess and pharyngeal spaces (parapharyngeal spaces, carotid spaces, retropharyngeal space) are considerably correlated. Quisy tonsillectomy is also considered as a therapy for peritonsillar abscess and repeated tonsillitis. Klebsiella pneumoniae, Staphylococcus aureus, and streptococci are common aerobic pathogens; Bacteroides and peptostreptococcus were the common nonaerobic pathogen. Surgical incision and drainage of deep neck abscess is warranted if empiric antibiotics cannot control the infectious condition. Disease progression often leads to sepsis and compromised airway condition. Diabetes mellitus, HIV infection, intravenous drug abuse, steroid therapy, chemotherapy, and other immune dysfunction diseases are common risk factors of DNI [1,2]. 

Obstructive sleep apnea syndrome (OSAS) is a sleep disorder featuring repeated episodes of apnea or hypopnea during sleep and intermittent arousals from sleep, which is a result of total and/or partial collapse of upper airways [3]. The disruption of breathing leads to intermittent hypoxemia and sympathetic activation [4,5], oxidative stress, systemic inflammation, and metabolic changes, resulting in cardiovascular and metabolic morbidities [5]. Current research showed that OSAS associated with known cardiovascular risk factors, such as obesity, insulin resistance, and dyslipidemia [6]. Antonopoulou et al. indicated that local and systemic inflammation relate to the pathophysiology of OSAS [7]. Inancli et al. also have found that OSAS associates with upper airway inflammation, and the inflammatory processes may be potential mediators of cardiovascular morbidity in these patients who have OSAS [8]. OSAS affects approximately 24% of men and 9% of women [9]. Obesity, male sex, and aging are major risk factors of OSAS [4]. 

Upper airway anatomy, such as airway length, lateral pharyngeal wall thickness, tongue volume and dilator muscle activity are important mechanisms that affect the occurrence of OSAS [4,10]. When it comes to surgical therapy for OSAS, uvulopalatopharyngoplasty (UPPP) combined with tonsillectomy is a common and widely performed surgery, which typically involves resection of the uvula, palatine tonsils, and posterior segment of the soft palate, remodeling the retropalatal airway and decreasing the collapsibility of the upper airway [11].

As stated before, many studies have addressed UPPP+ T (uvulopalatopharyngoplasty plus tonsillectomy) and its surgical response in the cardiovascular system [12,13]. Long-term complications of UPPP+ T include velopharyngeal insufficiency, pharyngeal symptoms, such as tightness or dryness, taste/voice disturbance, and nasopharyngeal stenosis [11]. However, the relationship between UPPP+ T and local infection, especially DNI, remains unclear. In our research, we compared the risk and severity of DNI in the patients with post-UPPP+ T to those without UPPP+ T. 

## 2. Materials and Methods

### 2.1. Data Source

In this cohort study, we used the data from the Taiwan National Health Insurance Research Database (NHIRD), which was established by the Taiwan government in 1995. The study period is from 2000 to 2012. The treatment combined UPPP and tonsillectomy is all performed between 2000 to 2007, the occurrence of DNI is observed after operation or till to end of 2012. We utilized the data derived from the Longitudinal Health Insurance Database (LHID) of the NHIRD in Taiwan from 1 January 2000, to 31 December 2012. This is because of the NHI authority only allowed us to analyze that time span and thus we could only survey that time period. Almost all of Taiwan's residents participated in the National Health Insurance program and the health information is stored in NHIRD. The information in the database includes medical services, devices, and prescription drug records. The diagnosis code recorded in the database followed the International Classification of Diseases, Ninth Revision, Clinical Modification (ICD-9-CM) code. The subset data, LHID, containing data of one million randomly selected insurances from the NHIRD, was the main data source. P.-C.H, L.-C.S., and Y.-A.T. collected the data and L.-C.S., Y.-A.T., C.-J.T., and C.-D.L. checked each datum and discussed the real DNI condition that was defined as an infection event. W.-D.C., J.-N.L., P.-S.L., H.-T.Y., and T.-C.S. rechecked the collected data and conducted the data analyses. This study was approved by the Institutional Review Board of China Medical University Hospital.

### 2.2. Study Population 

Our study was a case cohort study, considering patients who underwent UPPP+ T between 2000 and 2012. Those without UPPP+ T were the control patients. Four control patients were matched to a case patient by sex, age, index year, and comorbidities using propensity score matching. The index date of the case group was the date of received UPPP+ T and that of the control group was a random date within the study period. We eliminated patients developed outcomes of interest before the index date, with a follow-up time less than six months and all aged below 18. All subjects were followed until the occurrence of primary outcome, withdraw from the program, or the end of 2013.

### 2.3. Main Outcome and Comorbidities

Deep neck infection (DNI) (ICD-9-CM code 475, 478.22, 478.24 527.3, 528.3, 682.0, 682.1) was the primary outcome of this study. We excluded DNI that occurred within a month after UPPP+ T because physicians often prescribe post-operation antibiotics to prevent tonsillitis or DNI. Patients who had undergone UPPP+ T were selected using national health insurance (NHI) surgical order (66025B). The UPPP+ T is not only for treating sleep apnea or snoring, but also for repeated tonsillitis and chronic pharyngeal inflammation [14]. So, as for UPPP+ T subgroup analyzation, after excluding deviated nasal septum disease (ICD-9-CM code 470), other diseases of upper respiratory tract (ICD-9-CM code 478) and other sleep disturbances (ICD-9-CM code 780.59), we further divided UPPP+ T cohort into OSAS-caused group and inflammation/infection group. Obstructive sleep apnea syndrome (OSAS) was defined by ICD-9-CM code 780.51, 780.53, 780.57, 327.20, 327.23, 327.29, 327.8, 780.50, 786.09. Inflammation and infection group was defined by ICD-9-CM code 474.0, A315, 474.0, A319, 472.0, 463, 474.11. The procedures of UPPP+ T included tonsillectomy and pharyngoplasty. One of the indications of UPPP+ T is chronic tonsillitis with snoring [15]. Chronic tonsillitis means 3–4 occurrences of tonsillitis per year [16]. The palatine tonsils represent the nidus of inflammation, and therefore surgery, such as combined UPPP and tonsillectomy, involving the removal of infection source in the tonsil, could treat the tonsillar-related infection. UPPP+ T is not only used to treat chronic tonsillitis, but also breathing disorders like snoring. To clarify whether the risk of DNI increased after UPPP+ T, we analyzed the incidence rate of DNI after 1–60 months between the control group and UPPP+ T cohort. 

For further study, we also looked into the length of hospital stay, emergency department (ED) admission and stayed in intensive care unit (ICU) to determine the seriousness of DNI [17]. Airway complications such as intubation (NHI procedure code 47031C), tracheostomy (NHI procedure code 56022C) and oxygen inhalation (NHI procedure code 57003C and 57004C) were observed to assess the progression and severity for DNI. Diabetes mellitus, hypercholesterolemia, overweight and obesity, depression, hypertension, deviated nasal septum, nasal polyps, hypertrophy of tonsils and adenoids, asthma, and gastroesophageal reflux disease were considered as comorbidities of UPPP+ T.

### 2.4. Statistical Analysis

To examine the distribution of sex, age group, and comorbidities between the case cohort and the control cohort, the chi-square test was used. The average age among two groups was tested by the Student’s t-test. Univariable and multivariable Cox proportional hazard models were employed to estimate the crude hazard ratio (cHR) and the adjusted hazard ratio (aHR). The Kaplan–Meier method was applied to obtain the cumulative incidence curve and testing was performed using the log-rank test. Odds ratios were estimated by the logistic regression model, and the relationship of changes was analyzed using simple linear regression. All statistical analyses were conducted using R Statistical Software, version 3.5.2 and SAS software, version 9.4 (SAS Institute Inc., Cary, NC, USA).

## 3. Results

A total of 1574 patients with UPPP+ T and 6296 patients without UPPP+ T were recruited in our cohort study. Refer to Table 1, male patients were dominant in the participants. Most of them were in the 30–39 year-old age group and the mean age of the controls and cases were 40.3 and 38.7. The UPPP+ T patients had a higher proportion of hypertrophy of tonsils and adenoids than non-UPPP+ T patients.

Table 2 demonstrates the incidence and hazard ratio of baseline factors for DNI. Relative to those subjects aged 18–29, the DNI risk is higher in 30–39, 40–49, and over 50 age group, especially subjects aged 40–49, where the risk of DNI increased by 1.52 folds (95% CI = 1.01–2.30). Patients with diabetes mellitus will have an increased risk of DNI (aHR = 1.73; 95% CI = 1.12–2.67). 

In Table 3 and Table 4, we perform a subgroup analysis, which showed that patients with OSAS history constitute 76.1% (*n* = 1198) of the UPPP+ T cohort. Compared to the control group, there was no significantly increased incidence rate of DNI in UPPP+ T cohort within 1–60 months (*p* > 0.05), as shown in Table 3. The slope of the regression line for the incidence rate of DNI for UPPP+ T (r = −0.92, R^2^ = 0.45) and non-UPPP+ T (r = 0.08, R^2^ = 0.06) decreased over follow-up, indicating that incidence rate of DNI decreased over the course for UPPP+ T. The difference in the slope change of the incidence rate of DNI was statistically significant (*p* < 0.05). The incidence rate and hazard ratios of DNI between UPPP+ T subgroups have no significant difference (Table 4).

The mean hospital stay of the UPPP+ T cohort (5.20 days) was significantly shorter than non-UPPP+ T cohort (7.81 days), with an adjusted relative ratio of 0.60, and patients in the inflammation/infection group were not hospitalized (Table 5). In the non-UPPP+ T cohort, 4.41% patients (*n* = 6) were admitted from the emergency department (ED), 0.74% patients (*n* = 1) were transferred to the intensive care unit (ICU), while the UPPP+ T cohort had only 1.59% patients (*n* = 1) admitted from ED and no case transferred to ICU (Table 5). Considering non-UPPP+ T patients as the reference group, the UPPP+ T cohort had a lower intubation rate, with an adjusted odds ratio of 0.47 (95% CI = 0.32–0.69) (Table 6).

## 4. Discussion

**Figure 1:**
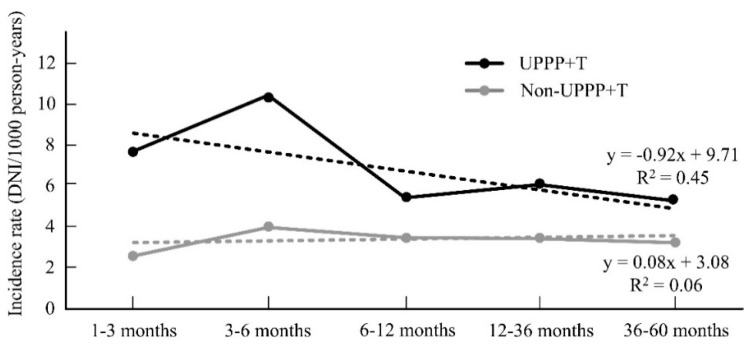
Changes in the slopes of DNI incidence rate.

The combined UPPP and tonsillectomy including tonsillectomy is indicated for sleep apnea and chronic tonsillitis with snoring. It is considered to cause an immune compromising condition due to the partial removal of immune tissue at the pharynx. However, the risk of deep neck infection is not significant after combined UPPP and tonsillectomy within 1–60 months. In addition, we found that combined UPPP and tonsillectomy not only reduces the severity for DNI by decreasing intubation rate and shortening the length of hospitalization. In comparison to non-UPPP+ T, the baseline of DNI incident rate was higher in UPPP+ T. In fact, the surgical procedure of combined UPPP and tonsillectomy includes tonsillectomy, uvulectomy, and suture palatoplasty. Therefore, the palatine tonsils are removed after combined UPPP and tonsillectomy. Thus, the possible tonsillitis is treated, decreasing the inflammatory condition caused by chronic tonsillitis. In addition, UPPP+ T is also adopted as a surgery for sleep apnea and intermittent hypoxic condition. It is possible that improved sleep apnea or less intermittent hypoxemia after UPPP+ T also rendered anti-inflammatory therapeutic results that reduced the incidence of deep neck infection. In general, the incidence rate is higher in the UPPP+ T group than non-UPPP+ T group. With time, there was a more prominent decrease in DNI incidence rate than in the non-UPPP+ T group, as shown in Figure 1.

### 4.1. Risk Factors for Deep Neck Infection

A review by Knapp et al. concluded that it is a common concept that diabetic mellitus (DM) patients can be infected more easily [18]. Moreover, it is known that DM is a common risk factor of DNI [1,2]. In line with previous studies, we found that the risk of DNI in the DM group was significantly higher than the non-DM group, with an adjusted hazard ratio of 1.73 (*p* < 0.05; Table 2). As stated before, odontogenic infection is the main cause of DNI. Adoviča et al. indicated that the occurrence rate of dental infection was significantly lower in elders because of the fewer teeth in their oral cavity [19]. Another study by Zamiri et al. also found that in the sixth, seventh, and eighth decades, the incidence rate of dental infection was lowest [20]. Our study had a similar result in that the adjusted hazard ratio was lower in the age group over 50 than age groups 40–49 and 30–39 (Table 2). However, contrary to the findings of previous research, our finding showed that compared to control group (age group 18–29), the DNI risk was higher in age group 30–39, 40–49, and over 50 (Table 2). Especially in age group 40–49, there was a significantly higher risk of DNI than the control group, with an adjusted hazard ratio of 1.52 (*p* < 0.05), as shown in Table 2. An apparent concern is that although the major infection source of DNI is dental infection, DNI presents less in elder patients, possible because they are more concerned about their teeth and have better hygiene. Besides, other infection sources exist, such as tonsillitis and pharyngitis. Furthermore, it is also important to consider the comorbidity in elder people. In the UPPP+ T group composing OSA that may also impact immunity, poor sleep quality leads to an immune compromising condition. There were also case of tonsillitis in the UPPP+ T group. In the combined UPPP and tonsillectomy, the tonsillectomy study illustrated that surgery has a protective effect in the case of cardiac problems, dementia, etc. [13,21,22] performed in Taiwan and Korea [23]. There were also studies showing the impact of adenotonsillectomy on the evolution of inflammatory markers that decreases tonsillitis related infection.

### 4.2. Risk of Deep Neck Infection after Uvulopalatopharyngoplasty

From our main result, we found that in patients with DNI, the patients without combined UPPP and tonsillectomy had a more consistent and lower incidence rate. However, compared to the control group, there was no significantly increased incidence rate of DNI after combined UPPP and tonsillectomy within 1–60 months (Table 3). This may imply that combined UPPP and tonsillectomy does not increase the risk of DNI within 1–60 months. Combined UPPP and tonsillectomy involves resection of the uvula, tonsils, and soft palate [11]. The tonsils provide the first line of protection against foreign pathogens, such as bacteria and viruses, but there is still a debate on whether tonsillectomy has a negative effect on the immune system [24]. We reviewed previous studies about the impact of immune system after tonsillectomy and two opposing opinions appeared (Table 7). Kaygusuz et al. found that long-term immune function shows no difference with healthy controls after tonsillectomy [25]. In a recent systematic review by Altwairqi et al., tonsillectomy had no negative affect on both humeral and cellular immunity in children and the level of immunoglobulin would recover to normal range postoperatively [26]. The other systematic review also showed that tonsillectomy has no negative clinical or immunological sequalae on the immune system [27]. A similar pattern of results was obtained in our study, showing that combined UPPP and tonsillectomy does not increase the risk of DNI within 1–60 months. 

In contrast, Wang et al. identified that the risk of DNI significantly increased among patients who have undergone tonsillectomy [24], and Duval et al. reported that adenotonsillectomy would change the humoral and cellular response of the immune system in children [28]. Another cohort study demonstrated that early-life tonsillectomy and adenoidectomy were associated with higher long-term risks of respiratory and infectious diseases [29]. However, very few publications mention the influence of tonsillectomy in adulthood. Whether tonsillectomy affects adult immune system still needs more investigation.

### 4.3. Adjustment of Obstructive Sleep Apnea Syndrome to Deep Neck Infection

Previous studies have indicated that the most common cause of DNI among adults is odontogenic or of salivary origin, such as dental and periodontal infection [1,2]. Normally, the oral biome is in balance, but mouth breathing in OSAS patients leads to dryness of the oral cavity and may also decrease the self-cleaning ability of the oral mucosa, leading to increased periodontal microbiota colonization and a chronic inflammatory response [30,31]. Xu et al. also revealed that changes of oxygen concentrations in the oral cavity in OSA patients might be associated with oral dysbiosis [32]. In a recent research by Ding et al. [33], there is a significantly higher occurrence of DNI in patients with sleep apnea. According to above research, there may be a correlation between DNI and OSAS. 

In our study, we found that the incidence rate of DNI between UPPP+ T and non-UPPP+ T cohort had no significant difference (Table 3), even though a higher OSAS composition of combined UPPP and tonsillectomy subgroup (76.1%) might link to higher hypoxia-related inflammation and DNI risk. The combined UPPP and tonsillectomy is the main scope of our study. Moreover, the direction of selecting the research object and experimental design were different at the beginning, possibly explaining the different results compared to the above studies. However, a larger sample size study is still warranted in the future.

### 4.4. Severity of Deep Neck Infection after Uvulopalatopharyngoplasty

Based on our result, we found that combined UPPP and tonsillectomy can reduce the length of hospitalization and intubation rate of DNI. Length of hospitalization is often used as an indicator of infection severity. As reported by Sakarya et al., the average hospital stay of DNI patients was 12.9 days [34]. A single-center analysis by Kauffmann et al. showed that the mean duration of DNI hospital stay was 15.3 days, and patients with diabetes mellitus had a significantly longer duration of hospitalization [35]. Another retrospective review conducted by University of Kentucky focused on the postoperative length of stay in patients with DNI, which showed the overall hospitalization period was three days [36]. These studies also indicated that age, comorbidity, such as diabetes mellitus, development of complications, and treatment only with medicine service led to longer hospital stay days [34,35,36]. From our result, in patients with DNI, the mean hospital stay of the UPPP+ T cohort (5.20 days) was significantly lower than the non-UPPP+ T cohort (7.81 days) (Table 5). Furthermore, in the non-UPPP+ T cohort, 4.41% patients (*n* = 6) were admitted from the emergency department (ED) and 0.74% patients (*n* = 1) transferred to intensive care unit (ICU), while the UPPP+ T cohort had only 1.59% patients (*n* = 1) admitted from ED and no case transferred to ICU (Table 5). This may imply that combined UPPP and tonsillectomy can reduce the severity of DNI. However, it must be pointed out that the duration of hospitalization of DNI in our study was shorter than most other research [34,35]. In Taiwan, it is very convenient for the public to seek medical treatment because of the implementation of Taiwan's health insurance system. Hence, people in Taiwan get medical intervention earlier before the diseases progress. 

We also observed airway complications, such as intubation, tracheostomy, and oxygen inhalation, to analyze the severity of DNI. Compared to the control group, the UPPP+ T cohort had a lower intubation rate (aOR = 0.47, *p* < 0.001; Table 6). Tracheostomy rate and oxygen using the rate of UPPP+ T cohort showed no significant difference compared to control group. Based on our result, it may imply that combined UPPP and tonsillectomy can reduce the intubation rate in DNI patients. 

### 4.5. Limitations

Our study had some limitations. First, this study used the data from the Taiwan National Health Insurance Database (NHIRD), which may be affected by regional characteristics and cannot fully represent the overall situation of the world. Moreover, Taiwan’s national health insurance allows people to get medical treatment regardless of their socio-economic status. Hence, some data may not be generalized to other populations. Second, the crucial data for surgical decision making, such as the apnea-hypopnea index and body mass index, are not available in our study, since NHIRD does not include biometric data of patients. Third, the incidence of multilevel combined UPPP and tonsillectomy increased from 2000 to 2012 in Taiwan [37]. Since NHIRD does not record patients’ medical records, and whether combined UPPP and tonsillectomy is combined with other palatal, nasal, hypopharyngeal, or tongue surgery, it may affect the subsequent incidence of DNI and complication rate. Fourth, length of hospitalization as a severity indicator of DNI, may vary from institution to institution because of different medical resources. Fifth, this study is the first cohort study to confirm the relationship between combined UPPP and tonsillectomy and DNI, so it is hard to compare our result to previous research. Furthermore, the number of patients referred from ED and admitted to ICU was too small. Hence, the data were just for information. Last, much research on tonsillectomy and its impact on immunity in children has been done, but previous publications rarely mention the influence of tonsillectomy in adulthood. Since our study excluded patients aged below 18, whether tonsillectomy affects the adult immune system still needs more investigation to clarify.

## 5. Conclusions

Our study investigated the association between combined UPPP and tonsillectomy and DNI. Based on our study, we identified that combined UPPP and tonsillectomy does not increase the risk of DNI within 1–60 months. Moreover, it may imply the efficacy of combined UPPP and tonsillectomy in reducing the severity of DNI by decreasing the intubation rate and length of hospitalization.

## Figures and Tables

**Table 1:** Baseline characteristics of patients.

	Non-UPPP+ T		UPPP+ T		
	No (*n* = 6296)		Yes (*n* = 1574)		
Variable	*n*	%	*n*	%	*p*-Value
Gender					0.76
female	1636	26.0	415	26.4	
male	4660	74.0	1159	73.6	
Age, year					0.87
18–29	1695	26.9	425	27.0	
30–39	1826	29.0	463	29.4	
40–49	1545	24.5	393	25.0	
≥50	1230	19.5	293	18.6	
mean, (SD)	40.26	(14.9)	38.74	(12.3)	<0.001
Comorbidities					
diabetes mellitus	700	11.1	180	11.4	0.72
hypercholesterolemia	692	11.0	168	10.7	0.72
overweight and obesity	189	3.0	46	2.9	0.87
depression	650	10.3	148	9.4	0.28
hypertension	1428	22.7	349	22.2	0.67
deviated nasal septum	1513	24.0	352	22.4	0.16
nasal polyps	96	1.5	27	1.7	0.59
hypertrophy of tonsils and adenoids	169	2.7	66	4.2	0.002
asthma	880	14.0	219	13.9	0.95
gastroesophageal reflux disease	141	2.2	35	2.2	0.97

n: number of patients; UPPP: uvulopalatopharyngoplasty; SD: standard deviation.

**Table 2:** Incidence and hazard ratio of baseline factors for Deep Neck Infection.

	Deep Neck Infection						
Variables	n	PY	IR	cHR	(95% CI)	aHRꝉ	(95% CI)
Gender							
Female	61	15,216	4.01	1.00	(reference)	1.00	(reference)
Male	138	39,428	3.50	0.87	(0.64, 1.18)	0.85	(0.63, 1.15)
Age, year							
18–29	42	15,664	2.68	1.00	(reference)	1.00	(reference)
30–39	63	16,342	3.86	1.45	(0.98, 2.15)	1.44	(0.97, 2.14)
40–49	58	13,876	4.18	1.58	(1.06, 2.35) *	1.52	(1.01, 2.30) *
≥50	36	8762	4.11	1.51	(0.97, 2.37)	1.38	(0.83, 2.27)
Comorbidities							
Diabetes mellitus							
No	170	50,049	3.40	1.00	(reference)	1.00	(reference)
Yes	29	4595	6.31	1.80	(1.21, 2.68) **	1.73	(1.12, 2.67) *
Hypercholesterolemia							
No	186	50,327	3.70	1.00	(reference)	1.00	(reference)
Yes	13	4317	3.01	0.78	(0.45, 1.38)	0.65	(0.36, 1.17)
Overweight and obesity							
No	194	53,589	3.62	1.00	(reference)	1.00	(reference)
Yes	5	1055	4.74	1.25	(0.51, 3.04)	1.05	(0.42, 2.6)
Depression							
No	180	50,440	3.57	1.00	(reference)	1.00	(reference)
Yes	19	4204	4.52	1.23	(0.76, 1.97)	1.15	(0.71, 1.85)
Hypertension							
No	157	45,060	3.48	1.00	(reference)	1.00	(reference)
Yes	42	9584	4.38	1.22	(0.86, 1.71)	1.08	(0.73, 1.6)
Deviated nasal septum							
No	165	44,173	3.74	1.00	(reference)	1.00	(reference)
Yes	34	10,471	3.25	0.84	(0.58, 1.22)	0.86	(0.59, 1.25)
Nasal polyps							
No	198	54,108	3.66	1.00	(reference)	1.00	(reference)
Yes	1	536	1.86	0.49	(0.07, 3.5)	0.54	(0.08, 3.94)
Hypertrophy of tonsils and adenoids							
No	193	53,440	3.61	1.00	(reference)	1.00	(reference)
Yes	6	1204	4.98	1.33	(0.59, 3.00)	1.31	(0.58, 2.98)
Asthma							
No	180	48,701	3.70	1.00	(reference)	1.00	(reference)
Yes	19	5943	3.20	0.84	(0.52, 1.34)	0.84	(0.52, 1.35)
Gastroesophageal reflux disease							
No	197	54,174	3.64	1.00	(reference)	1.00	(reference)
Yes	2	470	4.26	1.11	(0.27, 4.49)	1.04	(0.25, 4.23)

n: number of patients; PY: person-year; IR: incidence rate pre 1000 person-years; cHR: crude hazard ratio; aHR: adjusted hazard ratio; CI: confidence interval; ꝉ: adjusted by sex, age and comorbidities; *: *p*-value < 0.05; **: *p*-value < 0.01.

**Table 3:** Analysis of the incidence rate of DNI among the follow-up.

				Incidence rate of DNI per 1000 Person-Years				
	*n*	Event ^a^	Baseline	1–3 Months	3–6 Months	6–12 Months	12–36 Months	36–60 Months
Non-UPPP+ T	6296	136	2.16	2.56	3.96	3.46	3.44	3.23
UPPP+ T	1574	63	4.01	7.66	10.40	5.41	5.96	5.25
* p* value				0.15	0.23	0.54	0.18	0.46
OSAS cause	1198	41	3.42	10.07	10.27	5.39	4.48	6.61
*p* value				0.15	0.33	0.22	0.27	0.19
Inflammation and infection	195	7	3.58	0	0	0	1.92	0
*p* value				na	na	na	0.93	na

n: number of patients; DNI: deep neck infection; OSAS: Obstructive sleep apnea syndrome; UPPP: uvulopalatopharyngoplasty; na, not applicable; ^a^ number of DNI before the interventions; * *p* < 0.05, compare to baseline by using chi-square test.

**Table 4:** Incidences rate and hazard ratios of DNI between the UPPP+ T subgroups.

	n	Event ^a^	PY	Incidence Rate	Crude HR	Adjusted HR
UPPP+ T						
OSAS-caused	1198	41	8219	4.99	1 (reference)	1 (reference)
Inflammation and infection	195	7	1811	3.87	0.81 (0.36, 1.81)	0.82 (0.36, 1.85)

n: number of patients; PY: person-year; Crude HR: crude hazard ratio; Adjusted HR: adjusted hazard ratio; ^a^ number of DNI before the interventions.

**Table 5:** S Severity of DNI between UPPP+ T and non-UPPP+ T groups.

	Hospital Stay		ED	ICU
	Mean, (SD)	aRR (95% CI)	*n* (%)	*n* (%)
Non-UPPP+ T	7.81 (5.50)	1 (reference)	6 (4.41%)	1 (0.74%)
UPPP+ T	5.20 (1.81)	0.60 (0.43, 0.84) **	1 (1.59%)	0 (0%)
OSAS cause	5.40 (0.98)	0.62 (0.40, 0.96) *	0 (0%)	0 (0%)
Inflammation and infection	-	-	0 (0%)	0 (0%)

n: number of events; CI: confidence interval; SD: standard deviation; UPPP+ T: uvulopalatopharyngoplasty plus tonsillectomy; ED: emergency department admission; ICU: intensive care unit; DNI: deep neck infection; OSAS: Obstructive sleep apnea syndrome;.adjusted RR: adjusted by sex, age and comorbidities; *: *p*-value <0.05; **: *p*-value <0.01.

**Table 6:** Airway complication rate.

	Intubation		Tracheostomy		Oxygen Inhalation	
	*n* (%)	Adjusted OR (95% CI)	n (%)	Adjusted OR (95% CI)	*n* (%)	Adjusted OR (95% CI)
Non-UPPP+ T	251 (4.02%)	1.00 (reference)	31 (0.49%)	1.00 (reference)	720 (12.9%)	1.00 (reference)
UPPP+ T	31 (1.97%)	0.47 (0.32, 0.69) ***	13 (0.83%)	1.74 (0.90, 3.35)	188 (14.8%)	1.18 (0.98, 1.41)

n: number of events; OR: odds ratio; CI: confidence interval; UPPP+ T: uvulopalatopharyngoplasty plus tonsillectomy; adjusted OR: adjusted by sex, age and comorbidities; ***: *p*-value <0.001.

**Table 7:** Reviewed studies—impact of tonsillectomy on immune system.

Study	Year	Study Design	Main Results
Positive Opinion			
Kaygusuz et al. [25]	2009	cross-sectional	Tonsillectomy does not impair long-term (54 months) humoral and cellular immunity of children compared to their early-stage immune status (1 month). Moreover, the long-term (54 months) immune function has no different than healthy controls.
Bitar et al. [27]	2015	systematic review	Tonsillectomy has no negative clinical or immunological sequalae on the immune system.
Altwairqi et al. [26]	2020	systematic review	Tonsillectomy has no negative affect on both humeral and cellular immunity in children.
Negative Opinion			
Duval et al. [28]	2008	retrospective case-control study	Adenotonsillectomy would change the humoral and cellular response of the immune system in children.
Wang et al. [24]	2015	cohort study	Risk of DNI increased after tonsillectomy
Byars et al. [29]	2018	cohort study	Early-life (before age 9) tonsillectomy and adenoidectomy were associated with higher long-term (age 30) risks of respiratory, infectious, and allergic diseases.

## Data Availability

Data from the Taiwan National Health Insurance Research Database (NHIRD).
